# Continuous glucose monitoring in acute ischemic stroke patients treated with endovascular therapy: A pilot study to assess feasibility and accuracy

**DOI:** 10.1371/journal.pone.0280153

**Published:** 2023-02-09

**Authors:** C. J. B. A. Kersten, A. A. M. Zandbergen, M. J. Fokkert, R. J. Slingerland, H. M. den Hertog

**Affiliations:** 1 Department of Neurology, Medisch Spectrum Twente, Enschede, The Netherlands; 2 Department of Internal Medicine, Erasmus Medisch Centrum, Rotterdam, The Netherlands; 3 Department of Clinical Chemistry, Isala, Zwolle, The Netherlands; 4 Department of Neurology, Isala, Zwolle, The Netherlands; Stanford University School of Medicine, UNITED STATES

## Abstract

**Introduction:**

Hyperglycemia is common in acute ischemic stroke and is associated with larger infarct volume and unfavorable functional outcome, also in patients who undergo reperfusion therapy. Hyperglycemia during reperfusion may be a therapeutic target. However, previous randomized trials on the effect of glucose lowering in the acute phase of ischemic stroke failed to demonstrate effects on clinical outcome. Inaccurate glucose measurements and not focussing on patients who undergo reperfusion therapy are possible explanations. Our aim was to study the feasibility and accuracy of continuous glucose monitoring (CGM) in patients with acute ischemic stroke undergoing endovascular treatment (EVT).

**Methods:**

All consecutive patients with ischemic stroke and large vessel occlusion (LVO) of the anterior circulation who were eligible for endovascular therapy within 24 hours of symptom onset and presenting at the emergency department of Isala Hospital Zwolle, the Netherlands, were enrolled in this study. CGM was performed using a Freestyle Libre Flash 2 device (FSL-CGM, Abbot Diabetes Care, Alameda, California, USA) which was implanted on arrival at the emergency department. Feasibility was defined as the number of patients who could be registered for 24 hours and delay in door-to-groin time because of sensor implantation. Accuracy of CGM versus capillary and venous based plasma glucose values was determined with the Parkes error grid analysis.

**Results:**

Twenty-three patients were included of whom 20 completed 24 hours monitoring (87%). One patient did not give permission to use the data; one sensor broke during implantation and one meter was broken after a sensor was shot in so no measurements could be recorded. There was no significant delay in treatment due to implantation of the sensor and no adverse events. One hundred percent of CGM data are in zones A and B of the Parkes error grid analysis so data out of the sensor can be interpreted as accurate.

**Conclusion:**

In this study, we showed that continuous glucose monitoring in patients with acute ischemic stroke due to large vessel occlusion of the anterior circulation in patients who were treated with endovascular therapy is feasible, safe and accurate.

## Introduction

Hyperglycemia is common in acute ischemic stroke, with a prevalence up to 30–50% in patients without a history of diabetes mellitus [1–3]. Increased glucose levels have been associated with larger final infarct volume and worse clinical outcome. Also in patients who underwent intravenous thrombolysis and endovascular therapy, which are acute reperfusion therapies in ischemic stroke, this association has been demonstrated [4–9]. Restitution of blood flow within the first few hours after stroke onset can (partially) prohibit that potentially salvageable penumbral tissue is being converted in irreversible core [10]. This relationship is “time dependent”. In human neuronal networks exposed to hypoxia, significant changes in firing activity and synchronicity were present already one hour after onset of hypoxia [11].

Because, hyperglycemia seems to affect the penumbral tissue in particular, glucose lowering therapy might be an effective therapeutic target in the acute phase of ischemic stroke in patients who undergo reperfusion therapy. Several studies have assessed the effect of active lowering glucose without any effect on infarct size or favorable functional outcome [12–17]. Only one study started the intervention in the early phase of acute ischemic stroke (<6 hours after stroke symptoms). They failed to realise target glucose levels in the intervention group. Also, glucose levels were determined capillary by a single finger prick at the emergency department followed by a varying number of capillary glucose measurements, each with several hours in between during admission on the stroke unit. This late and inaccurate observations may have contributed to the lack of effect of glucose lowering therapy on outcome after acute stroke in previous studies.

Continuous glucose monitoring (CGM) could assess the evolution of glucose levels during acute ischemic stroke. Thereby, a glucose lowering intervention could be adapted to parallel continuous measurements. CGM-devices measure interstitial fluid glucose semi continuously with certain intervals. It is approved for outpatient use for diabetic patients without individual calibrating [18–20]. Earlier studies showed that also in the acute setting of the intensive care unit, CGM can be used safely and accurately [21]. Previous studies with CGM in stroke patients have not been performed in the acute phase and none of the studies focused on patients who underwent reperfusion therapy [22–25]. Particularly in this acute phase, CGM is interesting to be able to monitor fluctuations of glucose levels due to stress as well as the evolution of glucose levels during reperfusion therapies.

The aim of this pilot study was to assess the feasibility and accuracy of CGM in the first 24 hours of acute ischemic stroke in patients treated with endovascular therapy. This study is a design for a glucose lowering intervention study in which glucose levels can be continuously adjusted by means of these sensors.

## Methods

### Study design and patient selection

We conducted an observational pilot study in Isala Hospital Zwolle, The Netherlands. All consecutive patients with ischemic stroke and large vessel occlusion (LVO) of the anterior circulation who were eligible for endovascular therapy within 24 hours of symptom onset and presenting at the emergency department were enrolled in this study. Deferred consent was used and on during admission, informed consent was obtained from each patient or its legal representative. This pilot phase was used to test feasibility and the accuracy of CGM in this study population. Therefore, the sample size was small and feasibility was tested in 20 patients. The study protocol was approved by the medical ethics committee and the institutional research board of Isala Hospital Zwolle.

### Data collection

CGM was performed using a Freestyle Libre Flash 2 device (FSL-CGM, Abbot Diabetes Care, Alameda, California, USA). The FSL-CGM device was implanted by a trained physician by a single use applicator on arrival at the emergency department. Deferred consent was used. All patients were treated according to national and local stroke protocols (also with regard to hyperglycemia monitored during regularly blood tests). Notably, the alert option of the sensor for hypo- and hyperglycemia was not used because of our observational study design. Twenty-four hours after implantation, the sensor was removed.

Furthermore, we collected the following baseline data: demographic data, vascular risk factors and medical history, stroke severity assessed by means of the National Institutes of Health Stroke Scale (NIHSS), type of reperfusion therapy, time from onset to needle and/or groin puncture, admission plasma glucose (by capillary finger prick) and admission blood pressure. Glucose levels of >7.8 mmol/L were defined as hyperglycemic and systolic blood pressure > 185 mmHg and/or diastolic > 110 mmHg as hypertension on admission.

### Outcome measures and statistical analysis

Feasibility of using CGM in the acute phase was the main parameter in this pilot study. In addition, the accuracy of CGM in acute ischemic stroke was tested. Feasibility was defined as number of patients who complete 24 hours of glucose monitoring. We defined the use of CGM feasible when monitoring is successful for 24 hours in 75% of the included patients. Delay in the start of endovascular treatment due to implantation and adverse events were specifically assessed. Five minutes delay in start of endovascular treatment was determined as reasonable. Accuracy of CGM, was determined by parallel capillary finger prick at 08.00 AM; 09.00 AM;11.00 AM and 13.00 PM, independently of time of inclusion of the patient. Glucose values were also determined by venous measurements at these same moments. Capillary and venous measurements in plasma were determined with the Hexokinase method. According to previous literature the point accuracy of CGM versus capillary and venous based glucose values was determined with the Parkes error grid analysis [18, 26, 27]. Values in zones A and B are described as clinically acceptable. Values in zones C, D and E are determined as inaccurate. CGM is considered accurate when 95% of the CGM measurements are in zones A and B.

## Results

### Feasibility of CGM

Twenty-three patients were included of whom 20 completed 24 hours monitoring (87%). Characteristics of included patients are shown in Table 1. Mean age was 74 years (SD 16), 40% were men and median NIHSS was 7.5 (IQR 2.3–17.5). Median glucose on admission was 7.0 mmol/L (IQR 6.1–8.8), six patients (30%) were hyperglycemic on admission of whom 50% had a history of diabetes mellitus.

**Table 1:** Patient characteristics (N = 20).

Age in years, mean (SD)	74 (16)
Male sex, %	40
Current smoker, N (%)	11 (55)
Dyslipidemia*, N (%)	8 (40)
Glucose on admission, median (IQR)	7.0 (6.1–8.8)
Hyperglycemic, N (%)	6 (30)
Hypertension on admission**, N (%)	3 (15)
IVT treatment, N (%)	10 (50)
Onset to ED in minutes, median (IQR)	180 (105–675)
Presentation on ED to implantation sensor in minutes, median (IQR)	29 (23–35)

SD = standard deviation

*LDL-cholesterol >2,5 mmol/L; IQR = Interquartile Range

**Blood pressure >185/110 mmHg; IVT = thrombolysis; ED = Emergency Department.

Reasons for incomplete participation were: one patient did not give permission for use of the data; one sensor broke during implantation and the chip of one meter was no longer working so it registered no data. There was no significant delay in the start of endovascular treatment due to implantation of the sensor. No adverse events were assessed (no skin reactions for example).

### Accuracy of CGM and glucose evaluation

**Fig 1:**
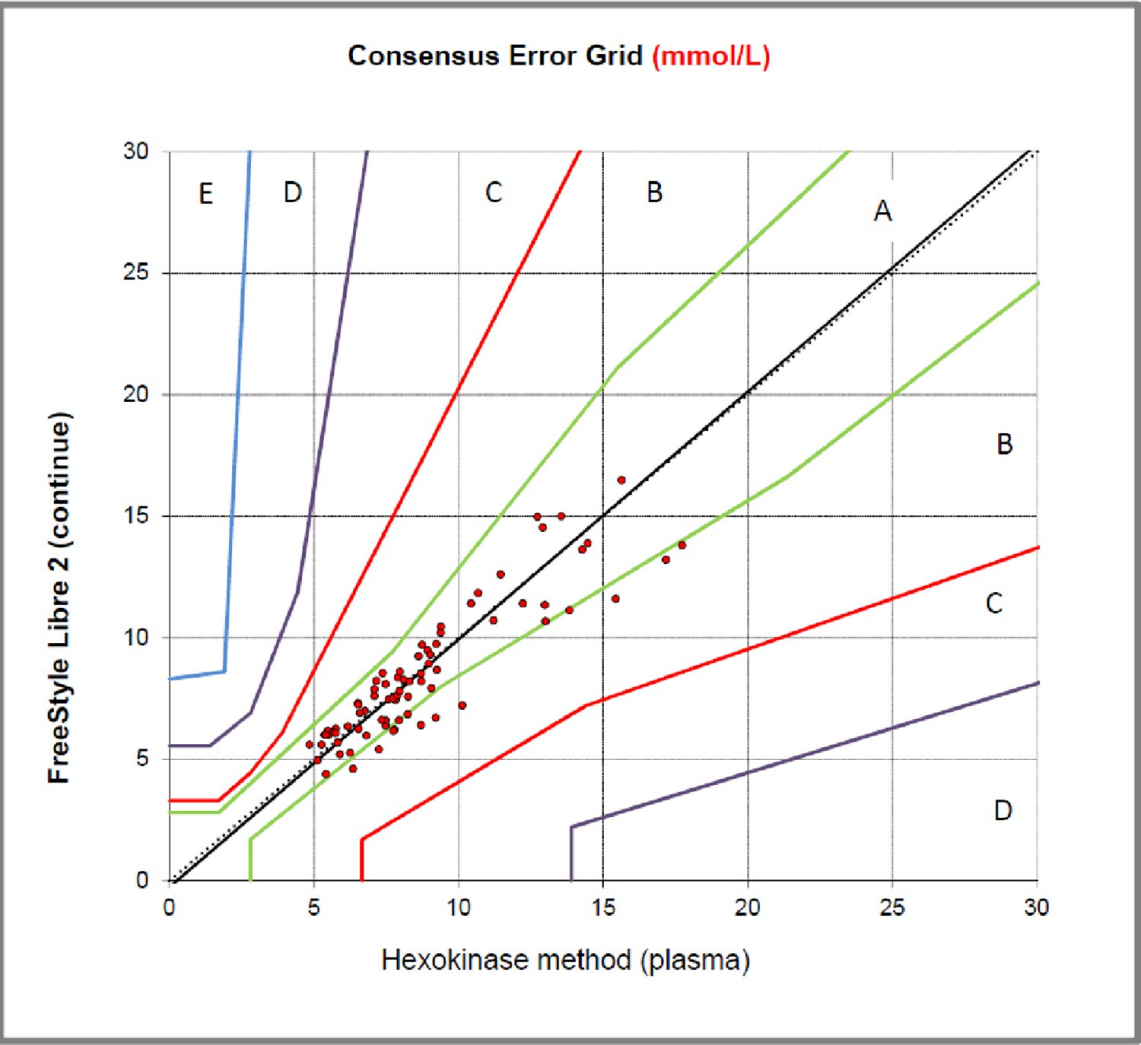
Parkes error grid of glucose levels with Freestyle Libre sensor versus capillary measurements. *Error grid of glucose levels with Freestyle Libre sensor versus venous measurements shows similar results.

One hundred percent of CGM data are in zones A and B of the Parkes error grid analysis, both for capillary and venous plasma Hexokinase reference measurements (Fig 1).

**Fig 2:**
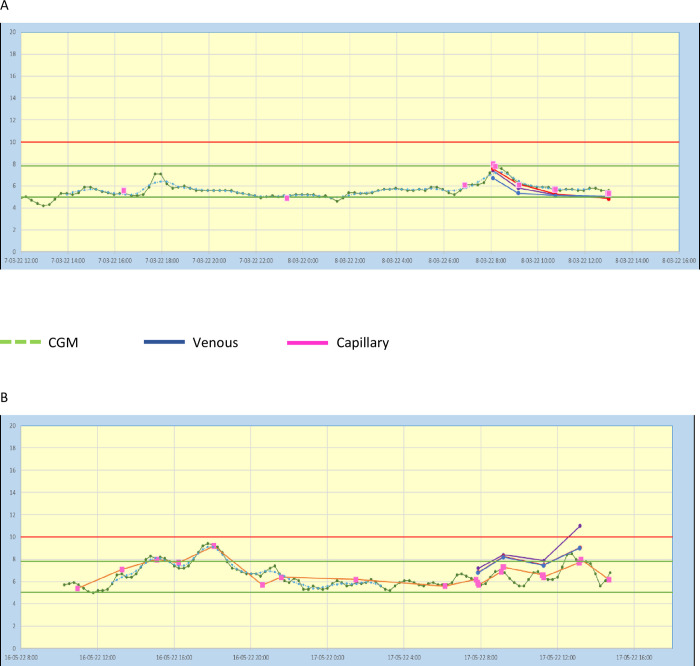
CGM data and capillary and venous glucose levels of a patient with glucose levels in normal range (A) and with hyperglycemia (B).

Particularly in normal glucose ranges (5–8 mmol/L) the sensor shows good accuracy. In the higher and lower glucose rates, we found some variation between data of the sensor and capillary and venous measurements, Fig 2.

## Discussion

In this pilot study, we found that continuous glucose monitoring in patients with acute ischemic stroke due to large vessel occlusion of the anterior circulation in patients who were treated with endovascular therapy is feasible, safe and accurate.

In outpatients with diabetes mellitus, CGM has been proven to be accurate, resulting in a significant decrease in HbA1c [18–20]. In line with our results, CGM was marked as a promising diagnostic tool in critically ill patients on an intensive care unit (ICU) [21]. In this study, glucose levels were monitored continuously for up to 72 hours in 22 patients with various diagnoses for which ICU treatment was indicated. However, none of these patients had acute ischemic stroke. CGM has been previously studied in patients with ischemic stroke [22–25]. In a study of 68 ischemic stroke patients, median time from stroke onset to first CGM measurement was 15 hours and most patients had small infarct volumes [22]. Admission glucose were 9.2 mmol/L (IQR 3.5–19.5) and 6.0 mmol/L (IQR 4.5–10.4) in patients with and without known diabetes mellitus. Glucose values reached a minimum at 14 hours after stroke onset in both groups. Another study with 100 participants included both patients with ischemic stroke and patients with intracerebral hemorrhage [25]. Many patients stayed hyperglycemic (glucose level >8 mmol/L) for at least 88 hours post-stroke in that study. Only one study applied CGM in patients who underwent endovascular therapy [24]. Persistent hyperglycemia was observed in 33% of included patients and infarct volume was significantly larger in the persistent hyperglycemic group. Nevertheless, also in these studies the first glucose measurement was eight hours after stroke onset.

Hundred percent of our data measured by the sensor and venous and capillary glucose levels fell within the accepted variation according to the Parkes error grid. Even though our data can be considered accurately, we have seen that the sensor underestimates the effect of meal on venous and capillary glucose levels. Particularly in the lower and higher glucose rates, we found some variation between the sensor and capillary and venous measurements. These variations are also described in previous literature [19].

Hyperglycemia on admission has been related to poor functional outcome in ischemic stroke, but previous studies showed no favorable effects of a glucose lowering intervention in the acute phase of stroke on outcome [12]. Possible reasons include inadequate glucose measurement as well as frequent occurrence of hypoglycemia. Besides hyperglycemia on admission, also the duration of high blood glucose levels are related to unfavorable outcome [25]. An area under the curve of more than 8 mmol/L of blood glucose and distribution time of more than 8 mmol/L of blood glucose have been described as the most influential factors related to unfavorable functional outcome. Possibly, a more specified intervention in the acute phase of ischemic stroke in which glucose measurements are much more accurately and can be adjusted continuously to actual glucose level, could be effective. Moreover, specific patient groups might benefit from a glucose lowering intervention in the acute phase of ischemic stroke. For example, stress hyperglycemia appears to particularly affect ischemic stroke patients without diabetes mellitus and the impact of hyperglycemia on functional outcome has been mainly described in patients with a large mismatch between ischemic core and penumbra and when early reperfusion is achieved [28]. To determine which patients might benefit in particular from a glucose lowering intervention, a larger observational study with continuous monitoring would be valuable to identify patients with the most disturbed glucose evolution. The observations from the present study show that CGM is a promising method to observe the evolution of glucose levels in acute ischemic stroke and to select categories of patients in which an intervention can be valuable.

The strength of our study is that this is the first study in which CGM is applied in the very early phase of acute ischemic stroke treated with endovascular therapy. Some limitations should be discussed. The sample size was small because this was a pilot study. Therefore we could not assess associations between glucose levels and functional and radiological outcomes. In addition, the sensor underestimates the effect of meals on blood glucose levels and shows a delay in glucose excursions because of transport time of glucose from blood to interstitial fluid. It turned out impossible to determine venous and capillary glucose samples strictly on 08.00, 09.00. 11.00 and 13.00 o’clock for example because patients had therapies or additional investigations. Because some venous and capillary measurements have a different interval to meals the variation of data out of the sensor in comparison with capillary and venous measurements in this study is possibly larger than it actually is.

In conclusion, we found that CGM in patients with acute ischemic stroke treated with endovascular therapy is feasible, safe and accurate. It is a promising method to observe the evolution of glucose levels during the acute phase of stroke and to monitor glucose levels strictly during a glucose lowering intervention study in the future.

## Supporting information

S1 Data (SAV)
